# Real-world outcomes of unfractionated heparin plus antithrombin concentrate versus bivalirudin in adult patients undergoing extracorporeal membrane oxygenation

**DOI:** 10.1051/ject/2026015

**Published:** 2026-09-15

**Authors:** Amanda Arenella, Jiani Zhou, Joshua Oh, Michael Calloway, Reuben Howden, Stephen O. Bader

**Affiliations:** 1 Grifols SSNA 79 T.W. Alexander Drive, Research Triangle Park NC 27709 USA; 2 University of North Carolina, 9201 University City Boulevard Charlotte NC 28223 USA; 3 Heritage Valley Health System 1000 Dutch Ridge Road Beaver PA 15009 USA

**Keywords:** Antithrombin III, Extracorporeal membrane oxygenation, Anticoagulants, Heparin, Coronary bypass, Health resources, Health care costs

## Abstract

*Background*: Extracorporeal membrane oxygenation (ECMO) provides life support to patients undergoing cardiopulmonary surgeries when other interventions are ineffective. Unfractionated heparin (UFH) is routinely used during ECMO due to the increased risk of thrombosis. However, in some cases, like antithrombin deficiency, UFH is not efficacious, and heparin resistance (HR) can develop. There is considerable confusion about HR and what additional management strategies should be taken. *Objectives*: This study compared clinical and economic outcomes associated with antithrombin concentrate (ATc) + UFH versus bivalirudin in adult patients undergoing ECMO. *Methods*: This retrospective cohort study used data from the 2014–2023 TriNetX database and the 2018–2021 Premier Healthcare Database. Adult patients treated with ATc + UFH or bivalirudin within 3 days of ECMO initiation who did not undergo cardiopulmonary bypass (CPB) or coronary artery bypass grafting (CABG) during their ECMO hospital stay were identified. Propensity score matching was performed to balance the groups. Thromboembolic events, bleeding events, heparin-induced thrombocytopenia, hospital mortality, length of stay, and total hospital cost were compared. *Results*: A total of 1601 patients (276 ATc + UFH, and 1352 bivalirudin) were identified from TriNetX, and 1592 (264 ATc + UFH, and 1528 bivalirudin) from Premier. ATc + UFH was associated with lower odds of thrombotic and bleeding events compared to bivalirudin only. Length of stay and total hospital costs trended lower for patients prescribed antithrombin and UFH. *Conclusions*: Antithrombin + UFH may be a preferable option for managing ECMO patients, particularly those with heparin resistance.

## Introduction

According to data from the Extracorporeal Life Support Organization (ELSO) Registry, the use of extracorporeal membrane oxygenation (ECMO) at its member sites increased from 3,262 in 2009 (850 adult, 1,154 pediatric, and 1,258 neonatal) to 21,896 uses in 2021 (17,975 adult, 2,394 pediatric, and 1,527 neonatal) [1]. When ECMO is used, concomitant anticoagulant therapy is required to prevent clot formation within the mechanical circuit, pump, and oxygenator. The administered therapy must create a precise balance of clot prevention in the circuits while minimizing the risk of bleeding in these patients. The risk of thrombosis during ECMO has been reported to vary from 10% to 46.1%, depending on patient age and the type of circuit used [2]. The paucity of clinical data from well-controlled studies on anticoagulant use during ECMO has led to institutions establishing varying standards of care when clotting occurs. Historically, unfractionated heparin (UFH) has been the anticoagulant of choice during ECMO [3]. The preference for UFH has largely been the result of doctor familiarity given its long-term use, low cost, ease of titration, and the availability of a reversal agent [4].

However, it is important to note that UFH does not act as a direct anticoagulant. It acts by binding with antithrombin III (AT; previously ATIII), so its dose-response relationship is dependent on the quality and quantity of circulating AT [5–7]. A complicating factor when UFH is used during ECMO is heparin resistance (HR). HR is defined as a failure to achieve a prespecified level of anticoagulation with an appropriate dose of UFH, commonly identified as greater than 35,000 units per day [8, 9]. The clinical definition of heparin resistance varies by institution and can be dependent on the anticoagulation target, choice of coagulation lab test, and the condition indicating UFH [9]. A decrease in AT levels will decrease the effectiveness of UFH in thrombin inhibition, and as such, antithrombin deficiency has been suggested as the major reason for HR [7, 10, 11]. Though not all patients experiencing HR will have low AT levels due to the potential of other risk factors [12, 13]. For example, a study by Spiess and colleagues noted that CPB patients with low AT levels had a 57% chance of developing HR during surgery [13].

A potential treatment to counteract resistance to UFH is the administration of an antithrombin concentrate (ATc). Based on the mechanism of action of UFH, AT levels should correlate with responsiveness to UFH. Several studies and surveys have observed the utilization of ATc for patients experiencing HR [12, 14, 15], with some findings that ATc is effective in resolving HR irrespective of low antithrombin levels [14]. Acquired AT deficiency can develop under several conditions, including, but not limited to, liver disease, malnutrition, nephrotic syndrome, inflammatory bowel disease, disseminated intravascular coagulation, plasmapheresis, and heparin administration [12, 16].

HR can jeopardize surgical management by increasing heparin requirements and the risk of thrombotic complications [17]. Specifically, there are data that suggest an association between HR and an increased risk of adverse clinical events during the perioperative period, as well as longer hospital stays [15, 17]. Since the most common treatments for HR are either increasing UFH dose, adding or replacing with AT from human plasma, or changing from UFH to a direct thrombin inhibitor (DTI) [8, 9], it is important to consider the relative merits of each. There have been multiple comparison studies conducted to evaluate the safety profiles of each treatment and assess various risks during ECMO. Particularly, outcomes such as thrombotic events, bleeding events, length of hospital stay, and mortality were all analyzed in patients receiving UFH or patients receiving bivalirudin [18–20].

Bivalirudin is a direct thrombin inhibitor (DTI) and an effective alternative to heparin in ECMO [21–30]. Despite concerns about bleeding without a specific antidote, there has been increasing interest in DTI use during ECMO, particularly in cases with HR or heparin-induced thrombocytopenia (HIT) [30–32]. HIT is a prothrombic state paradoxically accompanied by decreased platelet counts (>50%) that typically develops 5–10 days after initiation of heparin therapy. Due to the severity of this condition, clinical guidelines recommend the immediate discontinuation of all heparin products and the switch to alternative anticoagulants, such as bivalirudin [30, 31, 33].

The current study was designed to compare real-world evidence, via patient data, from two large real-world databases. Given the variability in published and institutional definitions of HR [8, 9], the condition was operationally defined in this analysis by identifying patients who received UFH but required escalation to either adjunct ATc or an alternative DTI early after ECMO initiation. Clinical outcomes, healthcare resource utilization, and costs were evaluated between patients treated with ATc + UFH versus bivalirudin during ECMO treatment. Bivalirudin was chosen as the comparator since its use closely mirrors ATc use (i.e., situations where AT levels are critically low or UFH is not achieving desired results) [34], and it is the most common comparator in prior ECMO anticoagulation studies [18–29, 35–38].

## Materials and methods

### Real-world data sources

Retrospective observational cohort studies were conducted using de-identified data from two sources, the Premier^®^ Healthcare Database (PHD) and TriNetX^®^ Dataworks USA. The PHD contains information on patient demographics, admission and discharge diagnoses, financial information, patient disposition, and discharge health status described from standard hospital discharge billing files from over 700 geographically diverse hospitals and health systems across the US [39]. TriNetX Dataworks USA is a federated research network including de-identified electronic health records (EHR) data for over 112 million patients from 64 US-based healthcare organizations. The database includes detailed information on patient demographics, diagnoses, procedures, medications, laboratory measurements, and genomics [40]. This study is exempt from Institutional Review Board review because the data are de-identified and Health Insurance Portability and Accountability Act-compliant.

### Study design

Adult (aged ≥18 years) patients meeting the eligibility criteria described below were identified in the PHD between January 2018 and December 2021 and TriNetX between January 2014 and December 2023. These patients were categorized into mutually exclusive treatment groups: the antithrombin concentrate (ATc) + UFH group and the bivalirudin group (Supplementary Figure S1 PHD, and Supplementary Figure S2 TriNetX). The ATc + UFH group comprised patients who also received UFH during their inpatient stays for ECMO and were administered ATc within ±3 days of ECMO initiation. This cohort was limited to patients who received antithrombin supplementation via ATc, not via fresh frozen plasma or other sources. The bivalirudin group consisted of patients who only received bivalirudin within ±3 days of ECMO initiation.

Patients who underwent coronary artery bypass graft (CABG) surgery or CPB, received both ATc and bivalirudin during their ECMO encounters, or had missing sex data, were excluded for matching purposes and to maintain data integrity. For the bivalirudin group, patients with evidence of a past medical history of HIT were also excluded. This was assumed in the ATc + UFH group, as patients with this history are no longer recommended to use heparin products. The diagnosis and procedure codes used to identify eligible patients are provided in Supplemental Table S1.

### Clinical and economic outcome measures

Thromboembolic events (pulmonary embolism and deep vein thrombosis), bleeding events, HIT, and hospital mortality were measured using dichotomous variables, with a value of 1 indicating occurrence during the ECMO hospitalization. The diagnosis and procedure codes used to identify the outcomes of interest are provided in Supplemental Table S2. Specifically, Supplemental Table S2 contains the ICD-10 codes used to determine bleeding and thrombosis events, as well as HIT. Hospital length of stay (LOS) was measured as a continuous variable defined as the number of days between the discharge date and admission date. Total hospital cost, including the cost of treatments, was defined as the overall expense incurred during the entire hospitalization period and was adjusted to 2021 USD.

EHR and healthcare claims data uniquely provide valuable insights into a patient’s journey during hospitalization, but they each have their specific strengths and limitations. The strength of EHR data sources, like TriNetX, is that they contain detailed, granular clinical information, capturing each service and treatment provided, their timing, and clinical response. EHR data capture diagnoses, symptoms, lab results, medication lists, and physician notes associated with a hospitalization episode. Thus, EHRs are well-suited for determining clinical outcomes associated with specific services and/or treatments. For this study, TriNetX was chosen to assess clinical outcomes due to its ability to exclude patients with a past medical history of HIT and ensure outcomes occurred after the interventions were given. However, EHR data sources are not well suited for determining the cost of treatment, and in fact, cost data are rarely captured.

On the other hand, in claims databases, the services and care captured during a health care visit or hospitalization are billed for reimbursement. Data would include the billable costs, price, and payment information for each service. This information provides good insights into the costs and utilization of healthcare particularly comparative costs of care associated with healthcare interventions and/or treatments. Claims data sources can best show the effectiveness of care or treatments in terms of short or long-term cost savings, but they are less useful regarding the clinical effectiveness of individual interventions and treatments. Therefore, the PHD cohort was used for reporting the length of hospital stay and total cost. As a result, the clinical outcomes were assessed using our EHR data source, TriNetX, while the economic cost outcomes were assessed using the claims data source, PHD.

## Statistical analysis

To enhance comparability in the analyses, the ATc + UFH and bivalirudin groups were balanced using 1:1 propensity score matching based on patient demographics (age, sex, race, geographic region), hospital characteristics (urban–rural location, bed number, and teaching status; PHD cohort only), comorbidities, and Charlson Comorbidity Index (CCI) scores to remove the influence of potential cofounders on the two groups. Specifically, a Greedy nearest neighbor matching algorithm was employed with a caliper width of 0.1 of the standard deviation of the propensity score logit. An absolute value of the standardized mean difference (SMD) less than 0.1 indicates that an acceptable balance between the two groups on individual patient and hospital characteristics was achieved [41].

Pre- and post-match differences in patient characteristics between the ATc + UFH and bivalirudin groups were assessed using *t*-tests for continuous variables and chi-squared tests for categorical variables.

Regression models were selected based on the distribution and nature of each outcome of interest. Binary clinical outcomes, such as thromboembolic events, bleeding events, HIT, and hospital mortality, were compared using logistic regression models. Given the right-skewed nature of count data for duration of care, LOS was compared using generalized linear models with a negative binomial distribution. The total cost associated with the hospital encounters was compared using generalized estimating equation models with a gamma distribution and log link. Unadjusted and adjusted odds ratios (OR) from logistic regressions and incidence rate ratios (IRR), derived from generalized linear models or generalized estimating equation analyses, with corresponding 95% confidence intervals (CIs) and p-values were reported. A two-tailed test with an alpha level set at 0.05 was utilized to determine statistical significance.

All statistical analyses were performed using SAS^®^ version 9.4 (SAS Institute Inc., Cary, NC, USA) and R (version 4.3.3, R Core Team 2024) [42]. Specifically, in TriNetX analyses, the propensity score matching was implemented using the MatchIt package [43].

## Results

### Baseline characteristics

There were 1,792 patients who met the eligibility criteria in the PHD cohort. Before matching, 264 received ATc + UFH, and 1,528 patients received bivalirudin (Supplementary Table S3). The difference in median age for the ATc + UFH and bivalirudin cohorts was non-significant at 48.6 ± 16.2 and 49.9 ± 14.1 years, respectively, as was the percentage of females, 30.7% and 33.5% in the ATc + UFH and bivalirudin groups, respectively.

Table 1 provides post-matching results for both cohorts. The PHD cohort was comprised of 528 patients (264 in each group), and the TriNetX cohort was comprised of 552 patients (276 in each group). Post-match, the largest percentage difference across all relevant outcomes (not including geographic regions) in both cohorts was 3.4% in the PHD data. ATc + UFH was used more often in those identifying as white. The largest regional cohort difference was in the TriNetX data, where the ATc + UFH group was given more (8.4% difference) in the Midwest, while bivalirudin was administered more in the South (9% difference). Except for the regional differences, in both cohorts, the SMD estimates were all below the acceptable <0.01 level on all relevant outcomes. Therefore, the post-matched cohorts were appropriately comparable, providing added confidence in any treatment effects found. Supplementary Table S3 contains pre-matching results for both cohorts.

**Table 1:** Comparison of baseline demographics and clinical characteristics of patients treated with ATc + UFH versus bivalirudin in both databases in post-match analyses.

	Post-match PHD				Post-match TriNetX			
	ATc + UFH	Bivalirudin	*p*-Value	SMD	ATc + UFH	Bivalirudin	*p*-Value	SMD
*N*	264	264			276	276		
*Demographics*								
*Age at index date (yr)*								
Mean (SD); median	48.6 (16.2); 50.0	47.9 (14.7); 50.0	<0.001	0.05	39.3 (20.4); 40.0	40 (18.5); 36.0	0.214	−0.04
Sex = female	81 (30.7%)	80 (30.3%)	0.925	0.01	90 (32.6%)	97 (35.1%)	0.589	−0.05
*Race*								
White	179 (67.8%)	170 (64.4%)	0.408	0.07	171 (62%)	171 (62%)	1.000	0.00
Black	34 (12.9%)	43 (16.3%)	0.267	−0.10	49 (17.8%)	50 (18.1%)	1.000	−0.01
Other	51 (19.3%)	51 (19.3%)	1.000	0.00	56 (20.3%)	55 (19.9%)	1.000	0.01
*Region*								
Midwest	42 (15.9%)	28 (10.6%)	0.072	0.15	67 (24.3%)	44 (15.9%)	0.019	0.21
Northeast	32 (12.1%)	38 (14.4%)	0.441	−0.23	22 (8%)	21 (7.6%)	1.000	0.01
South	157 (59.5%)	155 (58.7%)	0.860	−0.06	115 (41.7%)	140 (50.7%)	0.040	−0.18
West	33 (12.5%)	43 (16.2%)	0.215	0.02	57 (20.7%)	66 (23.9%)	0.413	−0.08
Unknown	0	0	NA	0.00	15 (5.4%)	5 (1.8%)	0.040	0.19
*Comorbidities*								
MI	52 (19.7%)	47 (17.8%)	0.577	0.05	59 (21.4%)	53 (19.2%)	0.597	0.05
CHF	150 (56.8%)	157 (59.5%)	0.537	−0.05	167 (60.5%)	160 (58%)	0.603	0.05
PVD	53 (20.1%)	56 (21.2%)	0.747	−0.03	105 (38%)	102 (37%)	0.860	0.02
CVD	39 (14.8%)	38 (14.4%)	0.902	0.01	74 (26.8%)	73 (26.4%)	1.000	0.01
Dementia	0	0	NA	0.00	0 (0%)	0 (0%)	NA	NA
COPD	61 (23.1%)	62 (23.5%)	0.918	−0.01	82 (29.7%)	79 (28.6%)	0.851	0.02
Rheumatic disease	8 (3%)	8 (3%)	1.000	0.00	13 (4.7%)	15 (5.4%)	0.846	−0.03
Peptic Ulcer disease	9 (3.4%)	13 (4.9%)	0.384	−0.08	12 (4.3%)	13 (4.7%)	1.000	−0.02
Mild Liver disease	18 (6.8%)	17 (6.4%)	0.861	0.01	38 (13.8%)	42 (15.2%)	0.717	−0.04
Diabetes, UC	65 (24.6%)	73 (27.7%)	0.428	−0.07	31 (11.2%)	31 (11.2%)	1.000	0.00
Diabetes, C	42 (15.9%)	50 (18.9%)	0.359	−0.08	42 (15.2%)	50 (18.1%)	0.424	−0.08
Paralysis	11 (4.2%)	7 (2.7%)	0.337	0.08	14 (5.1%)	20 (7.2%)	0.376	−0.09
Renal disease	59 (22.4%)	69 (26.1%)	0.310	−0.09	90 (32.6%)	87 (31.5%)	0.855	0.02
Malignant tumors	9 (3.4%)	12 (4.6%)	0.504	−0.07	25 (9.1%)	20 (7.2%)	0.534	0.07
Severe liver disease	7 (2.7%)	9 (3.4%)	0.612	−0.04	31 (11.2%)	25 (9.1%)	0.481	0.07
Solid tumor, Met.	3 (1.1%)	3 (1.1%)	1.000	0.00	4 (1.4%)	4 (1.4%)	1.000	0.00
AIDS/HIV	1 (0.4%)	0	1.000	0.06	4 (1.4%)	3 (1.1%)	1.000	0.03
*CCI score*								
Mean (SD); median	3.6 (2.8); 3.0	3.6 (2.9); 3.0	<0.001	−0.02	4.5 (3.5); 4.0	4.4 (3.7); 4.0	0.548	0.03
*Provider characteristics*								
Population = rural	27 (10.2%)	18 (6.8%)	0.161	0.04	NA	NA	NA	NA
Hospital = teaching	212 (80.3%)	208 (78.8%)	0.666	0.12	NA	NA	NA	NA
*Hospital size (number of beds)*								
1–199	2 (0.8%)	5 (1.9%)	0.450	−0.15	NA	NA	NA	NA
200–499	55 (20.8%)	62 (23.5%)	0.463	−0.07	NA	NA	NA	NA
≥500	207 (78.4%)	197 (74.6%)	0.305	0.10	NA	NA	NA	NA

PHD = Premier Healthcare Database; ATc = antithrombin III concentrate; UFH = unfractionated heparin; SMD = standardized mean difference; SD = standard deviation; MI = myocardial infarction; CHF = congestive heart failure; PVD = peripheral vascular disease; CVD = cerebrovascular disease; COPD = chronic obstructive pulmonary disease; UC = uncomplicated; C = complicated; Met = metastatic; CCI = Charlson Comorbidity Index; NA = not available.

### Clinical outcomes

**Figure 1:**
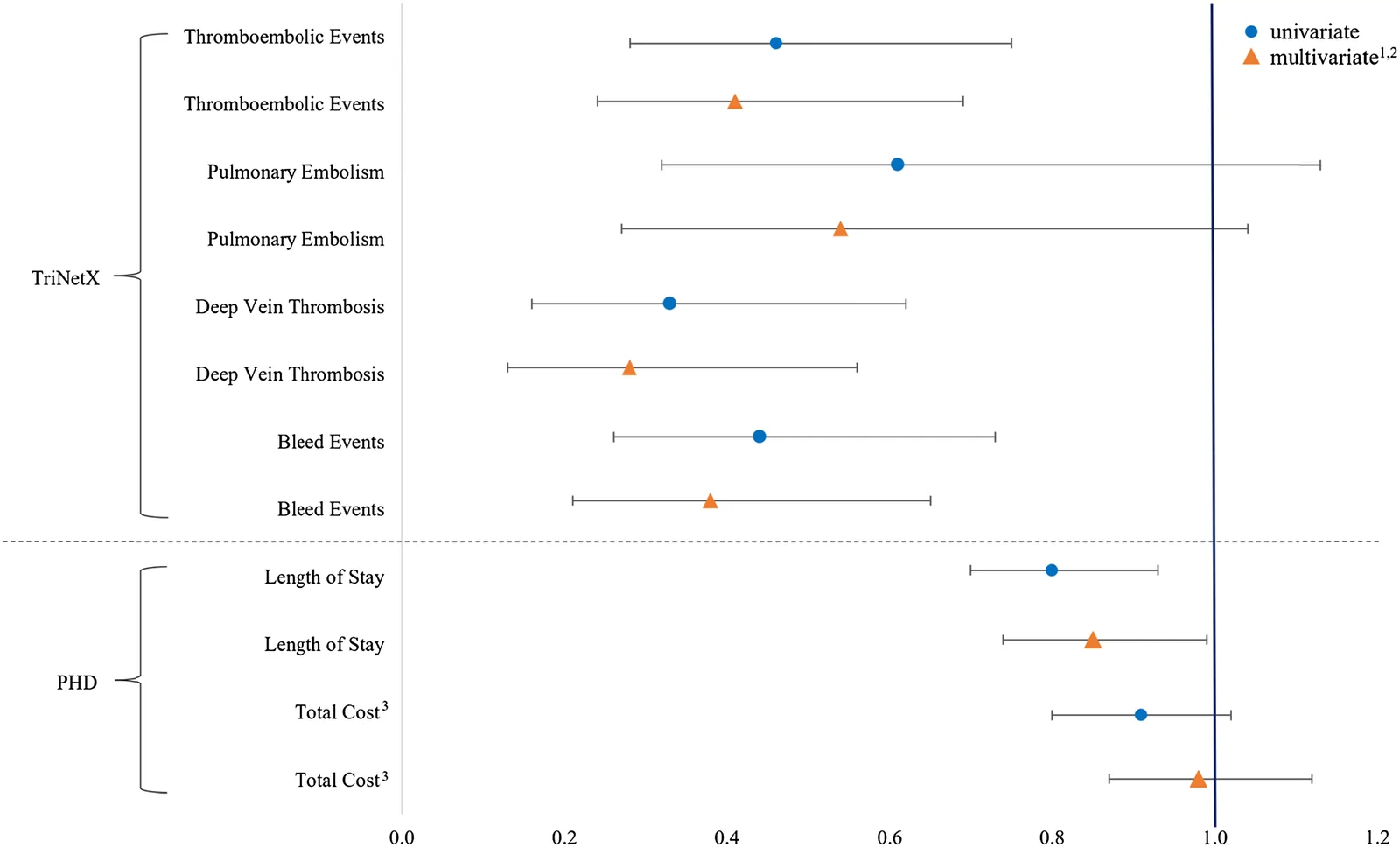
Forest plot of outcomes regression analysis for matched patients (ATc + UFH versus bivalirudin) in both databases. *X*-axis denotes odds ratios (OR) or incidence rate ratios (IRR) with the null significance indicated by the vertical line. *Y*-axis shows estimates for outcomes of interest from the TriNetX cohort (top) and the PHD cohort (bottom), Blue circles indicate unadjusted ORs or IRRs, and orange triangles indicate adjusted ORs or IRRs, with horizontal lines indicating 95% confidence intervals. Findings show that ATc + UFH is associated with lower odds of thrombotic and bleeding events as well as shorter hospital stays. ^1^Models adjusted for patient age, sex, race, geographic region, comorbidities, CCI score, and provider characteristics. ^2^Models adjusted for patient age, sex, race, geographic region, comorbidities, and CCI score. ^3^Total costs were adjusted for inflation and converted to 2021 USD. PHD = Premier Healthcare Database; ATc = antithrombin III concentrate; UFH = unfractionated heparin.

Table 2 shows the comparative treatment effects between the ATc + UFH and bivalirudin cohorts for both samples. In the TriNetX sample, 10.1% of ATc + UFH patients and 19.6% of bivalirudin patients experienced thrombosis (*p* = 0.003). Adjusted models for patient demographics and comorbidities showed that the use of ATc + UFH was associated with 59% lower odds of developing thrombosis during ECMO hospital stay compared to bivalirudin (adjusted OR: 0.41, 95% CI: 0.24–0.69) (Figure 1). The difference was largely driven by the reduction in the odds of developing deep vein thrombosis (adjusted OR: 0.28, 95% CI: 0.13–0.56).

**Table 2:** Comparison of clinical outcomes and resource utilization for matched patients (ATc + UFH versus bivalirudin) in both databases.

	ATc + UFH	Bivalirudin	*p*-Value
		**Post-match TriNetX (*n* = 276/group)**	
Thrombosis	28 (10.1%)	54 (19.6%)	0.003
Pulmonary embolism	17 (6.2%)	27 (9.8%)	0.157
Deep vein thrombosis	13 (4.7%)	36 (13%)	0.001
Bleed events	25 (9.1%)	51 (18.5%)	0.002
Heparin-induced thrombocytopenia	3 (1.1%)	6 (2.2%)	0.501
Mortality^1^	6 (2.2%)	3 (1.1%)	0.501
		**Post-match PHD (*n* = 264/group)**	
Length of stay, mean (SD); median	28.8 (29.4); 21	35.8 (31.6); 27	<0.001
Total cost, mean (SD); median^2^	$271,488 ($214,769); $212,079	$299,947 ($248,402); $229,727	0.3282

^1^Only the month and year of death are available. The date of death is assigned as the last day of the month of death.

^2^Total costs were adjusted for inflation and converted to 2021 USD.

PHD = Premier Healthcare Database; ATc = antithrombin III concentrate; UFH = unfractionated heparin; DTI = direct thrombin inhibitor; SD = standard deviation; NA = not available.

A similar pattern was observed for bleeding events. In the TriNetX sample, 9.1% of ATc + UFH patients and 18.5% of bivalirudin patients experienced bleeding events (*p* = 0.002) (Table 2). The adjusted model shows that patients treated with ATc + UFH had 62% lower odds of developing bleeding events compared to those treated with bivalirudin (adjusted OR: 0.38, 95% CI: 0.21–0.65) (Figure 1). The types of bleeding events observed are shown in Supplemental Table S2. Finally, less than 3% of patients died during their ECMO inpatient stays, and there was no difference in mortality between the ATc + UFH and bivalirudin groups.

### Healthcare resource utilization and cost outcomes

In the PHD sample, the average LOS for ATc + UFH patients and bivalirudin patients was 28.8 ± 29.4 days and 35.5 ± 30.3 days, respectively (*p* < .001). The difference remained statistically significant in the regression analysis after adjusting for the patient (age, sex, race, geographic region, comorbidities, and CCI score) and provider characteristics (adjusted IRR: 0.85; 95% CI: 0.74–0.99).

Lastly, the mean total costs for ATc + UFH and bivalirudin patients in the PHD were $271,488 (SD $214,769) and $299,947 (SD $248,402), respectively, with medians of $212,079 and $229,727, respectively (p = 0.328). Similarly, no statistical significance was observed after adjusting for patient and provider characteristics (adjusted IRR: 0.98; 95% CI: 0.87–1.12) (Figure 1).

## Discussion

UFH is the mainstay anticoagulant for ECMO. However, some patients develop resistance to UFH during ECMO. Treatment options for heparin resistance include ATc supplementation or switching from UFH to a DTI [8]. A recent survey found that these treatments are selected at roughly equal rates in practice as the primary alternative anticoagulant for HR [9]. AT is a critical component of UFH’s action to inhibit clotting [43, 44] while DTIs bind directly to thrombin, temporarily inhibiting the conversion of fibrinogen to fibrin [44–47]. To investigate which treatment option might best serve patients with heparin resistance, we compared real-world patient data from two large healthcare databases to assess the clinical outcomes, resource utilization, and costs for patients treated with ATc + UFH versus bivalirudin.

Several studies have been conducted that directly compared UFH with bivalirudin during ECMO [21–23, 25–29, 35–37]. Most of these retrospective studies found little difference in the anticoagulant effectiveness between UFH and bivalirudin [22, 25, 27, 28, 35, 37, 38]. In contrast, others reported an advantage for bivalirudin treatment in terms of the number of bleeding events [21, 23, 36], thrombosis [21], administration of fresh frozen plasma [21, 29, 36], platelets [23], and packed RBCs [21, 23, 36]. One study found a significant decrease in mortality in bivalirudin-treated compared to UFH-treated adult patients but not in pediatric patients [26].

Although multiple retrospective studies comparing UFH alone to bivalirudin alone have reported similar or favorable outcomes with bivalirudin in adults during ECMO [18–23, 35–37], the superiority of the ATc + UFH group in this study may reflect that it was not UFH alone, but UFH optimized with ATc supplementation. Given that AT deficiency can be common in ECMO [3, 9], restoring AT activity may improve heparin responsiveness and deliver more stable anticoagulation, thereby reducing both thrombotic complications and bleeding events [5, 7, 45]. Additionally, we specifically identified patients receiving ATc and UFH during the same encounter while also taking into consideration key potential confounding variables, such as history or incidence of HIT and those undergoing CPB. Variability in monitoring targets and dose titration across centers may have also influenced outcomes, as guidance for optimal monitoring in HR is not precise [3, 8, 15, 30].

Further, some studies investigated the economic and utilization impact of these clinical outcomes in real-world settings by comparing resource utilization. One study found that in pediatric patients, the total therapy costs were higher in the UFH-treated group than in the bivalirudin-treated group ($1,184/day versus $494/day; *p* = 0.03) [36] and another found that the total cost associated with hospital stay was higher in patients treated with bivalirudin (median $156,700) than in UFH-treated patients (median $144,900) [35].

Most of these studies did not report AT levels [21–25, 27, 28, 35, 37, 38], but those that did reported increased use of ATc in the UFH-treated groups [26, 29, 36]. A finding that is not surprising, given that AT is necessary for heparin’s anticoagulant activity, and the administration of exogenous ATc is a viable treatment for heparin resistance regardless of the ability to measure AT levels prior to administration. In addition, some of these studies included pediatric patients who have lower innate AT levels than adults [48, 49].

The differences among clinicians regarding what is considered to be HR are demonstrated by a survey of the members of The Perioperative and Critical Care Scientific Subcommittee of the International Society on Thrombosis and Haemostasis (ISTH) regarding the criteria for HR in medical and intensive care unit patients. The survey found that the dose of UFH each respondent considered excessive when trying to reach a monitoring goal was highly variable. Doses cited ranged from >10 to >60 units/kg/h or >35.000 to >50,000 units/day, while others answered that there is not a specific maximal dose [8]. Also, lab tests used to monitor the effects of UFH can confound the development of a consistent definition of HR. Tests can either be chromogenic or clot-based. Clot-based assays include activated partial thromboplastin time and activated clotting time, while chromogenic assays assess the activity of Factor Xa [8, 9].

In the current analysis that used two large, real-world data sources, there were some significant differences in patient characteristics between the two treatment groups. After the inclusion and exclusion criteria were met, 1,628 patients were found to have received either ATc + UFH or bivalirudin while receiving ECMO. The ATc + UFH cohorts were significantly younger than the bivalirudin cohort (Supplementary Table S3). The reason for this is not clear. The difference persisted post-matching in the PHD database (Table 1). In addition, there were some significant differences in the geographic distribution of patients between the groups in the pre-match analysis. These differences were not present post-matching.

In the patients from the TriNetX database, significantly higher percentages for some comorbidities (myocardial infarction, congestive heart failure, peripheral vascular disease, liver, and renal disease) and a higher mean CCI score were seen in the ATc + UFH group compared to the bivalirudin group before matching. These percentage differences were consistent with a higher prevalence in the ATc + UFH group, indicating that, in real-world use, ATc appears to be used more often in critically ill patients with more severe comorbidities due to acquired AT deficiency. Examples of conditions that are associated with acquired AT deficiency include acute and chronic liver disorders, which remain consistent with the pre- and post-match comorbidity demographic for severe liver disease in the ATc + UFH cohort [50]. This is supported by the finding that the ATc + UFH group had a significantly higher CCI score, but this difference was controlled for in the post-match groups.

This work has known limitations associated with real-world, retrospective data comprised of electronic health records or insurance claims. Most limitations relate to the capturing and/or recording of patient procedures and medications. Individual patient data can be sparse, incomplete, or incorrectly coded. In addition, biases can be created because of differing healthcare access, protocols governing treatments, and quality of providers and facilities. While medications prescribed can be captured, the extent of their compliance cannot. Additionally, the use of medications may be more a factor of convenience than the doctor’s preference.

One limitation of the study was that not all relevant outcomes were available in the retrospective claims data. For example, outcomes like extracorporeal cardiopulmonary resuscitation, the number of circuit changes, and identification of distal versus proximal deep vein thrombosis to determine the severity of the thrombotic event were not captured. Also, the timing of interventions could not be determined in the PHD data. Lastly, due to the rarity of the conditions being studied, relatively small cohorts were derived from these large databases because of the relatively infrequent use of ATc. Another limitation was the inability to define HR consistently to assess its prevalence in these patients. The distinction in practice between institutions further demonstrates the inconsistencies in defining HR, as there is no specific ICD-10 code for the condition.

Strengths of this study include the use of two large, longitudinal, real-world data sources that contributed 264 and 276 matched patients within the treatment cohorts. The data are broad in representing the geographical and site of care usage. Because ATc is only approved for use in hereditary antithrombin deficiency in the US, prescribing ATc in trauma-related events is rare. In addition, the TriNetX database includes timed interventions, which means only those thromboses and bleeding events that occurred after ATc + UFH or bivalirudin administration were analyzed. While specialized registries, such as the Extracorporeal Life Support Organization (ELSO) registry, provide unparalleled depth regarding ECMO-specific clinical parameters and complications, they lack the high-resolution pharmacy data necessary to accurately measure anticoagulation utilization. The real-world databases used in this study offer the granularity required for this pharmacological analysis. However, this highlights an opportunity for future registry evolution, where incorporating detailed pharmacy fields into specialized registries like ELSO would better facilitate large-scale comparative effectiveness research.

## Conclusions

This study provides valuable insights into the comparative clinical, resource utilization and cost-effectiveness of ATc combined with UFH versus bivalirudin in ECMO patients. The findings suggest that ATc + UFH is associated with lower odds of thrombotic and bleeding events as well as shorter hospital stays, despite being used in sicker patients demographically. Additionally, total hospital costs generally trended lower for patients treated with ATc + UFH.

The results suggest that ATc + UFH may be a preferable anticoagulation strategy, particularly for patients with heparin resistance or thrombotic risk. However, the study's limitations, including its retrospective design and potential biases, highlight the need for further prospective studies to confirm these findings and guide clinical practice. Both ATc + UFH and bivalirudin remain viable options, with treatment decisions tailored to individual patient needs and clinical circumstances. Future research should focus on refining anticoagulation strategies to improve patient outcomes in this critical care setting.

## Data Availability

The data used for this study are available from TriNetX and Premier Healthcare.

## Supplementary material

*Supplemental Table S1*: Diagnosis and procedure codes for identifying study cohort. *Supplemental Table S2*: Diagnosis and procedure codes for identifying outcomes of interest. *Supplemental Table S3*: Comparison of baseline demographics and clinical characteristics of patients treated with ATc + UFH versus bivalirudin in both databases in pre-match analyses. *Supplementary Figure S1*: Study cohort attrition for Premier Healthcare Database. ATc = antithrombin III concentrate, PHD = Premier Healthcare Database; DTI = direct thrombin inhbitor; CPB = cardiopulmonary bypass; ECMO = extracorporeal membrane oxygenation; UFH = unfractionated heparin; CABG = coronary artery bypass grafting. *Supplementary Figure S2*: Study cohort attrition for TriNetX. ATc = antithrombin III concentrate, PHD = Premier Healthcare Database; DTI = direct thrombin inhbitor; CPB = cardiopulmonary bypass; ECMO = extracorporeal membrane oxygenation; UFH = unfractionated heparin; CABG = coronary artery bypass grafting. Supplementary material of this article is available at https://ject.edpsciences.org/10.1051/ject/2026015/olm.
