# Supplementary material for: Real-world outcomes of unfractionated heparin plus antithrombin concentrate versus bivalirudin in adult patients undergoing extracorporeal membrane oxygenation
Source: J Extra Corpor Technol. 2026 Sep 15;58(3):237–45. doi: 10.1051/ject/2026015 (PMC13577646; doi:10.1051/ject/2026015)
Supplement: Supplementary file 1 [file ject-58-237-s1.zip › Supplemental Table S1.docx]

**Supplemental Table S1: Diagnosis and procedure codes for identifying study cohort**

| **Code** | **Code Type** | **Description** | **Comment** |
| --- | --- | --- | --- |
| 39.65 | ICD-9-CM | Extracorporeal membrane oxygenation (ECMO) | ECMO |
| 5A1522G | ICD-10-CM | Extracorporeal Oxygenation, Membrane, Peripheral Veno-arterial | ECMO |
| 5A1522H | ICD-10-CM | Extracorporeal Oxygenation, Membrane, Peripheral Veno-venous | ECMO |
| 33946 | CPT | Extracorporeal membrane oxygenation (ECMO)/extracorporeal life support (ECLS) provided by physician; initiation, veno-venous | ECMO |
| 33947 | CPT | Extracorporeal membrane oxygenation (ECMO)/extracorporeal life support (ECLS) provided by physician; initiation, veno-arterial | ECMO |
| 33948 | CPT | Extracorporeal membrane oxygenation (ECMO)/extracorporeal life support (ECLS) provided by physician; daily management, each day, veno-venous | ECMO |
| 33949 | CPT | Extracorporeal membrane oxygenation (ECMO)/extracorporeal life support (ECLS) provided by physician; daily management, each day, veno-arterial | ECMO |
| 33951 | CPT | Extracorporeal membrane oxygenation (ECMO)/extracorporeal life support (ECLS) provided by physician; insertion of peripheral (arterial and/or venous) cannula(e), percutaneous, birth through 5 years of age (includes fluoroscopic guidance, when performed) | ECMO |
| 33952 | CPT | Extracorporeal membrane oxygenation (ECMO)/extracorporeal life support (ECLS) provided by physician; insertion of peripheral (arterial and/or venous) cannula(e), percutaneous, 6 years and older (includes fluoroscopic guidance, when performed) | ECMO |
| 33953 | CPT | Extracorporeal membrane oxygenation (ECMO)/extracorporeal life support (ECLS) provided by physician; insertion of peripheral (arterial and/or venous) cannula(e), open, birth through 5 years of age | ECMO |
| 33954 | CPT | Extracorporeal membrane oxygenation (ECMO)/extracorporeal life support (ECLS) provided by physician; insertion of peripheral (arterial and/or venous) cannula(e), open, 6 years and older | ECMO |
| 33955 | CPT | Extracorporeal membrane oxygenation (ECMO)/extracorporeal life support (ECLS) provided by physician; insertion of central cannula(e) by sternotomy or thoracotomy, birth through 5 years of age | ECMO |
| 33956 | CPT | Extracorporeal membrane oxygenation (ECMO)/extracorporeal life support (ECLS) provided by physician; insertion of central cannula(e) by sternotomy or thoracotomy, 6 years and older | ECMO |
| 33957 | CPT | Extracorporeal membrane oxygenation (ECMO)/extracorporeal life support (ECLS) provided by physician; reposition peripheral (arterial and/or venous) cannula(e), CPT percutaneous, birth through 5 years of age (includes fluoroscopic guidance, when performed) | ECMO |
| 33958 | CPT | Extracorporeal membrane oxygenation (ECMO)/extracorporeal life support (ECLS) provided by physician; reposition peripheral (arterial and/or venous) cannula(e), percutaneous, 6 years and older (includes fluoroscopic guidance, when performed) | ECMO |
| 33959 | CPT | Extracorporeal membrane oxygenation (ECMO)/extracorporeal life support (ECLS) provided by physician; reposition peripheral (arterial and/or venous) cannula(e), open, birth through 5 years of age (includes fluoroscopic guidance, when performed) | ECMO |
| 33962 | CPT | Extracorporeal membrane oxygenation (ECMO)/extracorporeal life support (ECLS) provided by physician; reposition peripheral (arterial and/or venous) cannula(e), open, 6 years and older (includes fluoroscopic guidance, when performed) | ECMO |
| 33963 | CPT | Extracorporeal membrane oxygenation (ECMO)/extracorporeal life support (ECLS) provided by physician; reposition of central cannula(e) by sternotomy or thoracotomy, birth through 5 years of age (includes fluoroscopic guidance, when performed) | ECMO |
| 33964 | CPT | Extracorporeal membrane oxygenation (ECMO)/extracorporeal life support (ECLS) provided by physician; reposition central cannula(e) by sternotomy or thoracotomy, 6 years and older (includes fluoroscopic guidance, when performed) | ECMO |
| 33965 | CPT | Extracorporeal membrane oxygenation (ECMO)/extracorporeal life support (ECLS) provided by physician; removal of peripheral (arterial and/or venous) cannula(e), percutaneous, birth through 5 years of age | ECMO |
| 33966 | CPT | Extracorporeal membrane oxygenation (ECMO)/extracorporeal life support (ECLS) provided by physician; removal of peripheral (arterial and/or venous) cannula(e), percutaneous, 6 years and older | ECMO |
| 33969 | CPT | Extracorporeal membrane oxygenation (ECMO)/extracorporeal life support (ECLS) provided by physician; removal of peripheral (arterial and/or venous) cannula(e), open, birth through 5 years of age | ECMO |
| 33984 | CPT | Extracorporeal membrane oxygenation (ECMO)/extracorporeal life support (ECLS) provided by physician; removal of peripheral (arterial and/or venous) cannula(e), open, 6 years and older | ECMO |
| 33985 | CPT | Extracorporeal membrane oxygenation (ECMO)/extracorporeal life support (ECLS) provided by physician; removal of central cannula(e) by sternotomy or thoracotomy, birth through 5 years of age | ECMO |
| 33986 | CPT | Extracorporeal membrane oxygenation (ECMO)/extracorporeal life support (ECLS) provided by physician; removal of central cannula(e) by sternotomy or thoracotomy, 6 years and older | ECMO |
| 33987 | CPT | Arterial exposure with creation of graft conduit (eg, chimney graft) to facilitate arterial perfusion for ECMO/ECLS (List separately in addition to code for primary procedure) | ECMO |
| 33988 | CPT | Insertion of left heart vent by thoracic incision (eg, sternotomy, thoracotomy) for ECMO/ECLS | ECMO |
| 33989 | CPT | Removal of left heart vent by thoracic incision (eg, sternotomy, thoracotomy) for ECMO/ECLS | ECMO |
| 5A15 | ICD-10-PCS | Physiological Systems / Performance / Circulator | ECMO |
| 1022227 | CPT | Extracorporeal membrane oxygenation (ECMO)/extracorporeal life support (ECLS) provided by physician | ECMO |
| 233573008 | SNOMED | Extracorporeal membrane oxygenation | ECMO |
| 786451004 | SNOMED | Venoarterial extracorporeal membrane oxygenation | ECMO |
| 232969009 | SNOMED | Cardiac support using extracorporeal membrane oxygenation circuitry | ECMO |
| 60819 | RxNorm | Bivalirudin | DTI |
| J0583 | HCPCS | Injection, bivalirudin, 1 mg | DTI |
| 15202 | HCPCS | Argatroban | DTI |
| J0883 | HCPCS | Injection, argatroban, 1 mg (for non-esrd use) | DTI |
| J0891 | HCPCS | Injection, argatroban (accord), not therapeutically equivalent to J0883 1 mg (for non-esrd use) | DTI |
| J0898 | HCPCS | Injection, argatroban (auromedics), not therapeutically equivalent to I0883 1 mg (for non-esrd use) | DTI |
| 5224 | RxNorm | Heparin [Generic OR Unknown Brand | Heparin |
| OMOP5031264 | RxNorm | Heparin Sodium | Heparin |
| J1644 | HCPCS | Injection, heparin sodium, per 1000 units | Heparin |
| J7196 | HCPCS | Injection, antithrombin recombinant, 50 i.u. | ATc |
| J7197 | HCPCS | Antithrombin iii (human), per i.u. | ATc |
| 1009 | RxNorm | Antithrombin III | ATc |
| 33120 | CPT | Excision of intracardiac tumor, resection with cardiopulmonary bypass | CPB |
| 33305 | CPT | Repair of cardiac wound; with cardiopulmonary bypass | CPB |
| 33315 | CPT | Cardiotomy, exploratory (includes removal of foreign body, atrial or ventricular thrombus); with cardiopulmonary bypass | CPB |
| 33322 | CPT | Suture repair of aorta or great vessels; with cardiopulmonary bypass | CPB |
| 33335 | CPT | Insertion of graft, aorta or great vessels; with cardiopulmonary bypass | CPB |
| 33405 | CPT | Replacement, aortic valve, open, with cardiopulmonary bypass; with prosthetic valve other than homograft or stentless valve | CPB |
| 33406 | CPT | Replacement, aortic valve, open, with cardiopulmonary bypass; with allograft valve (freehand) | CPB |
| 33410 | CPT | Replacement, aortic valve, open, with cardiopulmonary bypass; with stentless tissue valve | CPB |
| 33422 | CPT | Valvotomy, mitral valve; open heart, with cardiopulmonary bypass | CPB |
| 33425 | CPT | Valvuloplasty, mitral valve, with cardiopulmonary bypass | CPB |
| 33426 | CPT | Valvuloplasty, mitral valve, with cardiopulmonary bypass; with prosthetic ring | CPB |
| 33427 | CPT | Valvuloplasty, mitral valve, with cardiopulmonary bypass; radical reconstruction, with or without ring | CPB |
| 33430 | CPT | Replacement, mitral valve, with cardiopulmonary bypass | CPB |
| 33460 | CPT | Valvectomy, tricuspid valve, with cardiopulmonary bypass | CPB |
| 33465 | CPT | Replacement, tricuspid valve, with cardiopulmonary bypass | CPB |
| 33474 | CPT | Valvotomy, pulmonary valve, open heart, with cardiopulmonary bypass | CPB |
| 33496 | CPT | Repair of non-structural prosthetic valve dysfunction with cardiopulmonary bypass (separate procedure) | CPB |
| 33500 | CPT | Repair of coronary arteriovenous or arteriocardiac chamber fistula; with cardiopulmonary bypass | CPB |
| 33504 | CPT | Repair of anomalous coronary artery from pulmonary artery origin; by graft, with cardiopulmonary bypass | CPB |
| 33641 | CPT | Repair atrial septal defect, secundum, with cardiopulmonary bypass, with or without patch | CPB |
| 33702 | CPT | Repair sinus of Valsalva fistula, with cardiopulmonary bypass | CPB |
| 33710 | CPT | Repair sinus of Valsalva fistula, with cardiopulmonary bypass; with repair of ventricular septal defect | CPB |
| 33720 | CPT | Repair sinus of Valsalva aneurysm, with cardiopulmonary bypass | CPB |
| 33736 | CPT | Atrial septectomy or septostomy; open heart with cardiopulmonary bypass | CPB |
| 33814 | CPT | Obliteration of aortopulmonary septal defect; with cardiopulmonary bypass | CPB |
| 33853 | CPT | Repair of hypoplastic or interrupted aortic arch using autogenous or prosthetic material; with cardiopulmonary bypass | CPB |
| 33860 | CPT | Ascending aorta graft, with cardiopulmonary bypass, includes valve suspension, when performed | CPB |
| 33864 | CPT | Ascending aorta graft, with cardiopulmonary bypass with valve suspension, with coronary reconstruction and valve-sparing aortic root remodeling (eg, David Procedure, Yacoub Procedure) | CPB |
| 33870 | CPT | Transverse arch graft, with cardiopulmonary bypass | CPB |
| 33910 | CPT | Pulmonary artery embolectomy; with cardiopulmonary bypass | CPB |
| 33916 | CPT | Pulmonary endarterectomy, with or without embolectomy, with cardiopulmonary bypass | CPB |
| 33922 | CPT | Transection of pulmonary artery with cardiopulmonary bypass | CPB |
| 33926 | CPT | Repair of pulmonary artery arborization anomalies by unifocalization; with cardiopulmonary bypass | CPB |
| 33533 | CPT | Coronary artery bypass, using arterial graft(s); single arterial graft | CABG |
| 33534 | CPT | Coronary artery bypass, using arterial graft(s); two arterial grafts | CABG |
| 33535 | CPT | Coronary artery bypass, using arterial graft(s); three arterial grafts | CABG |
| 33536 | CPT | Coronary artery bypass graft procedure involving four or more arterial grafts | CABG |
| 33510 | CPT | Coronary artery bypass, vein only; single coronary venous graft | CABG |
| 33511 | CPT | Coronary artery bypass, vein only; two coronary venous grafts | CABG |
| 33512 | CPT | Coronary artery bypass, vein only; two coronary venous grafts | CABG |
| 33513 | CPT | Coronary artery bypass, vein only; three coronary venous grafts | CABG |
| 33514 | CPT | Coronary artery bypass, vein only; four coronary venous grafts | CABG |
| 33515 | CPT | Coronary artery bypass, vein only; five coronary venous grafts | CABG |
| 33516 | CPT | Coronary artery bypass, vein only; six or more coronary venous grafts | CABG |
| 33517 | CPT | Coronary artery bypass, vein and arterial graft(s); single | CABG |
| 33518 | CPT | Coronary artery bypass, vein and arterial graft(s); two | CABG |
| 33519 | CPT | Coronary artery bypass, vein and arterial graft(s); three | CABG |
| 33521 | CPT | Coronary artery bypass, vein and arterial graft(s); four | CABG |
| 33522 | CPT | Coronary artery bypass, vein and arterial graft(s); five | CABG |
| 3523 | CPT | Coronary artery bypass, vein and arterial graft(s); six or more | CABG |
