# Supplementary material for: Real-world outcomes of unfractionated heparin plus antithrombin concentrate versus bivalirudin in adult patients undergoing extracorporeal membrane oxygenation
Source: J Extra Corpor Technol. 2026 Sep 15;58(3):237–45. doi: 10.1051/ject/2026015 (PMC13577646; doi:10.1051/ject/2026015)
Supplement: Supplementary file 1 [file ject-58-237-s1.zip › Supplemental Table S2.docx]

**Supplemental Table S2: Diagnosis and procedure codes for identifying outcomes of interest**

| **Code** | **Code Type** | **Description** | **Comment** |
| --- | --- | --- | --- |
| I60 | ICD-10-CM | Nontraumatic subarachnoid hemorrhage | Bleeding |
| I61.1 | ICD-10-CM | Nontraumatic intracerebral hemorrhage in hemisphere, cortical | Bleeding |
| I61.2 | ICD-10-CM | Nontraumatic intracerebral hemorrhage in hemisphere, unspecified | Bleeding |
| I61.3 | ICD-10-CM | Nontraumatic intracerebral hemorrhage in brain stem | Bleeding |
| J94. 2 | ICD-10-CM | Hemothorax | Bleeding |
| K90.2 | ICD-10-CM | Blind loop syndrome, not elsewhere classified | Bleeding |
| K92.1 | ICD-10-CM | Melena | Bleeding |
| K92.2 | ICD-10-CM | Gastrointestinal hemorrhage, unspecified | Bleeding |
| D75.82 | ICD-10-CM | Heparin induced thrombocytopenia (HIT) | HIT |
| I26 | ICD-10-CM | Pulmonary embolism | Thrombosis |
| I80.1 | ICD-10-CM | Phlebitis and thrombophlebitis of femoral vein | Thrombosis |
| I80.2 | ICD-10-CM | Phlebitis and thrombophlebitis of other and unspecified deep vessels of lower extremities | Thrombosis |
| I80.3 | ICD-10-CM | Phlebitis and thrombophlebitis of lower extremities, unspecified | Thrombosis |
| I80.8 | ICD-10-CM | Phlebitis and thrombophlebitis of other sites | Thrombosis |
| I80.9 | ICD-10-CM | I80.9 | Thrombosis |
| I81 | ICD-10-CM | Portal vein thrombosis | Thrombosis |
| I82.3 | ICD-10-CM | Embolism and thrombosis of renal vein | Thrombosis |
| I82.4 | ICD-10-CM | Acute embolism and thrombosis of deep veins of lower extremity | Thrombosis |

Note: Corresponding ICD-9-CM codes were used as well.
