# Supplementary material for: Real-world outcomes of unfractionated heparin plus antithrombin concentrate versus bivalirudin in adult patients undergoing extracorporeal membrane oxygenation
Source: J Extra Corpor Technol. 2026 Sep 15;58(3):237–45. doi: 10.1051/ject/2026015 (PMC13577646; doi:10.1051/ject/2026015)
Supplement: Supplementary file 1 [file ject-58-237-s1.zip › Supplemental Table S3.docx]

**Supplemental Table S3: Comparison of baseline demographics and clinical characteristics of patients treated with ATc + UFH versus bivalirudin in both databases in pre-match analyses.**

|  | **Pre-Match PHD** | | | | **Pre-Match TriNetX** | | | |
| --- | --- | --- | --- | --- | --- | --- | --- | --- |
|  | ATc+UFH | Bivalirudin | p-value | SMD | ATc+UFH | Bivalirudin | p-value | SMD |
| ***N*** | 264 | 1,528 |  |  | 276 | 1,352 |  |  |
| ***Demographics*** |  |  |  |  |  |  |  |  |
| ***Age at Index Date (yr)*** | | | | | | | | |
| **Mean (SD); Median** | 48.6 (16.2); 50.0 | 49.9 (14.1); 51.0 | < 0.001 | -0.09 | 39.3 (20.4); 40.0 | 50.3 (16.1); 53.0 | < 0.001 | -0.66 |
| **Sex = Female** | 81 (30.7%) | 512 (33.5%) | 0.368 | -0.06 | 90 (32.6%) | 462 (34.2%) | 0.667 | -0.03 |
| ***Race*** | | | | | | | | |
| **White** | 179 (67.8%) | 971 (63.6%) | 0.183 | 0.09 | 171 (62%) | 818 (60.5%) | 0.702 | 0.03 |
| **Black** | 34 (12.9%) | 218 (14.3%) | 0.549 | -0.04 | 49 (17.8%) | 191 (14.1%) | 0.146 | 0.10 |
| **Other** | 51 (19.3%) | 339 (22.2%) | 0.297 | -0.07 | 56 (20.3%) | 343 (25.4%) | 0.087 | -0.12 |
| ***Region*** | | | | | | | | |
| **Midwest** | 42 (15.9%) | 177 (11.6%) | 0.048 | 0.13 | 67 (24.3%) | 179 (13.2%) | < 0.001 | 0.31 |
| **Northeast** | 32 (12.1%) | 317 (20.8%) | 0.001 | -0.23 | 22 (8%) | 151 (11.2%) | 0.143 | -0.10 |
| **South** | 157 (59.5%) | 815 (53.3%) | 0.065 | 0.14 | 115 (41.7%) | 687 (50.8%) | 0.007 | -0.18 |
| **West** | 33 (12.5%) | 219 (14.3%) | 0.429 | 0.12 | 57 (20.7%) | 326 (24.1%) | 0.247 | -0.08 |
| **Unknown** | 0 | 0 | NA | 0.00 | 15 (5.4%) | 9 (0.7%) | < 0.001 | 0.40 |
| ***Comorbidities*** | | | | | | | | |
| **MI** | 52 (19.7%) | 346 (22.6%) | 0.287 | -0.07 | 59 (21.4%) | 305 (22.6%) | 0.726 | -0.03 |
| **CHF** | 150 (56.8%) | 744 (48.7%) | 0.015 | 0.16 | 167 (60.5%) | 564 (41.7%) | < 0.001 | 0.38 |
| **PVD** | 53 (20.1%) | 226 (14.8%) | 0.029 | 0.14 | 105 (38%) | 348 (25.7%) | < 0.001 | 0.28 |
| **CVD** | 39 (14.8%) | 185 (12.1%) | 0.227 | 0.08 | 74 (26.8%) | 184 (13.6%) | < 0.001 | 0.36 |
| **Dementia** | 0 | 3 (0.2%) | 1.000 | -0.06 | 0 (0%) | 4 (0.3%) | 0.812 | -0.06 |
| **COPD** | 61 (23.1%) | 331 (21.7%) | 0.600 | 0.03 | 82 (29.7%) | 307 (22.7%) | 0.016 | 0.16 |
| **Rheumatic Disease** | 8 (3%) | 41 (2.7%) | 0.750 | 0.02 | 13 (4.7%) | 42 (3.1%) | 0.246 | 0.09 |
| **Peptic Ulcer Disease** | 9 (3.4%) | 70 (4.6%) | 0.392 | -0.06 | 12 (4.3%) | 55 (4.1%) | 0.963 | 0.01 |
| **Mild Liver Disease** | 18 (6.8%) | 129 (8.4%) | 0.375 | -0.06 | 38 (13.8%) | 128 (9.5%) | 0.041 | 0.14 |
| **Diabetes, UC** | 65 (24.6%) | 452 (29.6%) | 0.101 | -0.11 | 31 (11.2%) | 159 (11.8%) | 0.884 | -0.02 |
| **Diabetes, C** | 42 (15.9%) | 235 (15.4%) | 0.826 | 0.01 | 42 (15.2%) | 181 (13.4%) | 0.478 | 0.05 |
| **Paralysis** | 11 (4.2%) | 57 (3.7%) | 0.732 | 0.02 | 14 (5.1%) | 51 (3.8%) | 0.403 | 0.07 |
| **Renal Disease** | 59 (22.4%) | 360 (23.6%) | 0.668 | -0.03 | 90 (32.6%) | 268 (19.8%) | < 0.001 | 0.31 |
| **Malignant Tumors** | 9 (3.4%) | 38 (2.5%) | 0.387 | 0.05 | 25 (9.1%) | 51 (3.8%) | < 0.001 | 0.25 |
| **Severe Liver Disease** | 7 (2.7%) | 50 (3.3%) | 0.596 | -0.04 | 31 (11.2%) | 41 (3%) | < 0.001 | 0.40 |
| **Solid Tumor, Met** | 3 (1.1%) | 11 (0.7%) | 0.447 | 0.04 | 4 (1.4%) | 18 (1.3%) | 1.000 | 0.01 |
| **AIDS/HIV** | 1 (0.4%) | 7 (0.5%) | 1.000 | -0.01 | 4 (1.4%) | 5 (0.4%) | 0.079 | 0.15 |
| ***CCI Score*** | | | | | | | | |
| **Mean (SD); Median** | 3.6 (2.8); 3.0 | 3.5 (2.8); 3.0 | < 0.001 | 0.04 | 4.5 (3.5); 4.0 | 3.6 (3); 3.0 | < 0.001 | 0.30 |
| ***Provider Characteristics*** | | | | | | | | |
| **Population = Rural** | 27 (10.2%) | 123 (8.1%) | 0.238 | -0.05 | NA | NA | NA | NA |
| **Hospital = Teaching** | 212 (80.3%) | 1254 (82.1%) | 0.492 | 0.08 | NA | NA | NA | NA |
| ***Hospital Bed Size*** | | | | | | | | |
| **1-199** | 2 (0.8%) | 7 (0.5%) | 0.629 | 0.04 | NA | NA | NA | NA |
| **200-499** | 55 (20.8%) | 265 (17.3%) | 0.172 | 0.09 | NA | NA | NA | NA |
| **≥500** | 207 (78.4%) | 1256 (82.2%) | 0.142 | -0.10 | NA | NA | NA | NA |

PHD **=** Premier Healthcare Database; ATc = antithrombin III concentrate; UFH = unfractionated heparin; SMD = standardized mean difference; SD = standard deviation; MI = myocardial infarction; CHF = congestive heart failure; PVD = peripheral vascular disease; CVD = cerebrovascular disease; COPD = chronic obstructive pulmonary disease; UC = uncomplicated; C = complicated; Met = metastatic; CCI = Charlson Comorbidity Index; NA = not available.
