# Supplementary material for: Real-world outcomes of unfractionated heparin plus antithrombin concentrate versus bivalirudin in adult patients undergoing extracorporeal membrane oxygenation
Source: J Extra Corpor Technol. 2026 Sep 15;58(3):237–45. doi: 10.1051/ject/2026015 (PMC13577646; doi:10.1051/ject/2026015)
Supplement: Supplementary file 1 [file ject-58-237-s1.zip › Supplementary Figure S1.docx]

**Supplementary Figure S1. Study cohort attrition for Premier Healthcare Database**

**ATc = antithrombin III concentrate, PHD = Premier Healthcare Database; DTI = direct thrombin inhbitor; CPB = cardiopulmonary bypass; ECMO = extracorporeal membrane oxygenation; UFH = unfractionated heparin; CABG = coronary artery bypass grafting.**
